# Comparison of Postoperative Breast Asymmetry Using Vectra 3D Imaging in Prepectoral Versus Subpectoral Implant-Based Breast Reconstruction

**DOI:** 10.3390/jcm13237486

**Published:** 2024-12-09

**Authors:** Seung-Ho Choi, Sang-Oh Lee, Kyu-Jin Chung, Il-Kug Kim, Jun-Ho Lee

**Affiliations:** Department of Plastic and Reconstructive Surgery, Yeungnam University College of Medicine, Daegu 42415, Republic of Korea; tmdgh9898@yu.ac.kr (S.-H.C.); lso915@naver.com (S.-O.L.); guzy7@hanmail.net (K.-J.C.)

**Keywords:** breast reconstruction, breast implant, three-dimensional imaging, postoperative complication, outcome assessment

## Abstract

**Background/Objectives:** Implant-based breast reconstruction (IBBR) is increasingly favored over autologous reconstruction due to its procedural simplicity and recovery benefits. Conducting this reconstruction using either the subpectoral or prepectoral planes has varied aesthetic outcomes. This study utilizes VECTRA XT 3D imaging to objectively assess breast symmetry differences between these surgical techniques. **Methods:** A retrospective cohort study was conducted analyzing data from patients undergoing unilateral total mastectomy followed by immediate silicone implant reconstruction via subpectoral or prepectoral techniques. The VECTRA XT 3D system provided measurements, including sternal-notch-to-nipple (SN-N), midline-to-nipple (ML-N), and nipple-to-inframammary fold (N-IMF) distances, as well as breast width, volume, and projection, taken more than a year postoperatively, to assess symmetry and aesthetic outcomes. **Results:** The study included 63 patients—29 in the subpectoral group and 38 in the prepectoral group. The SN-N ratio was 0.91 for the subpectoral group compared to 0.95 for the prepectoral group (*p* = 0.014). Among patients with a BMI of 25 or higher, the prepectoral group had an SN-N ratio significantly closer to 1 (0.97 ± 0.07) than the subpectoral group (0.89 ± 0.06) (*p* = 0.027). No statistically significant differences were found in metrics based on the surgical method across age categories divided at 50. **Conclusions:** The prepectoral IBBR technique shows improved nipple positioning and breast symmetry compared to subpectoral methods, as assessed via precise 3D imaging. This finding suggests potential advantages for surgical planning and patient satisfaction, indicating the need for large cohort studies to further investigate the factors influencing breast symmetry.

## 1. Introduction

The preference for implant-based breast reconstruction (IBBR) over autologous reconstruction post-mastectomy is increasing due to its procedural simplicity, reduced surgery duration, and expedited recovery period. In addition, acellular dermal matrix (ADM) is commonly used in IBBR after mastectomy to create a more natural breast contour [1,2].

Immediate breast reconstruction (IBBR) encompasses several techniques characterized by their coverage strategies using muscle. Total muscle coverage involves utilizing the pectoralis major muscle, supplemented laterally by either the serratus anterior muscle or its fascia. In contrast, partial muscle coverage, described as either subpectoral or dual plane, integrates partial use of the pectoralis major. Lastly, the prepectoral approach represents a strategy with no muscular coverage, utilizing the subcutaneous space for the placement of reconstructive materials [3]. Total muscle coverage can lead to suboptimal breast shape, insufficient inferior pole projection, and painful tissue expansion [4,5,6]. In contrast, partial muscle coverage, which involves the release of inferior pole constriction of the pectoralis muscle and the integration of acellular dermal matrix (ADM), enhances the breast contour and offers solid soft tissue coverage over the superior pole, albeit with a potential risk of minor animation deformity [7,8,9]. Recently, enhancements in the preservation of mastectomy skin flaps have contributed to the increasing acceptance of the prepectoral technique, wherein ADM is employed to cover implants using only the overlying soft tissue [6,10,11,12,13]. Although this approach presents challenges such as the high cost of ADM and potential issues related to implant rippling, palpability, and visibility in the superior pole, it offers significant benefits, including shorter surgical times and the promotion of a more natural breast shape. Furthermore, the use of ADM has been associated with reduced complications such as capsular contracture and improved patient satisfaction, positively impacting overall quality of life following surgery [14,15,16]. Such enhancements underline the importance of ADM in achieving satisfactory aesthetic outcomes and facilitating a smoother recovery for patients undergoing IBBR.

For patients undergoing breast reconstruction surgery, symmetry is one of the key indicators of postoperative satisfaction [17]. Therefore, accurately and objectively measuring asymmetry is crucial for evaluating cosmetic results and guiding subsequent surgical planning [18,19,20]. Naturally, breasts are inherently asymmetrical, and achieving perfect symmetry is often not possible. Even after IBBR, postoperative asymmetry is commonly observed [21,22]. Although scales such as the Harvard Cosmesis Scale or BREAST-Q™ are widely known for evaluating breast asymmetry, they have limitations and no gold standard tool currently exists [23,24]. VECTRA XT 3D, which uses multiple cameras to create a three-dimensional (3D) structure, offers more objective measurements of projection and volume than 2D photography [25]. A previous study found VECTRA XT 3D was superior in reliability and reproducibility, showing better inter- and intra-observer agreement [24].

Extensive research has compared the outcomes of subpectoral and prepectoral techniques, highlighting key aspects such as pain reduction, recovery time, capsular contracture rates, stability, and patient-reported outcomes. Notably, the prepectoral approach offers significant advantages, including reduced pain and quicker recovery, contributing to enhanced patient satisfaction and better overall postoperative experiences [26,27,28,29,30,31]. However, despite these insights, limited studies have objectively assessed long-term breast asymmetry related to potential implant malposition, capsular contracture, and shape changes over time between the two methods. Consequently, this study aims to employ VECTRA XT 3D technology to accurately measure and compare long-term breast asymmetry following direct-to-implant (DTI) breast reconstruction with subpectoral and prepectoral approaches.

## 2. Materials and Methods

### 2.1. Study Design

This single-institution, retrospective study was approved by the Institutional Review Board of Yeungnam University Hospital (IRB No. 2024-03-015) and conducted following the Declaration of Helsinki. Before acquiring VECTRA XT 3D scan images (Canfield Scientific, Parsippany, NJ, USA), informed consent was secured from all participants, ensuring compliance with ethical research protocols.

The study included patients who underwent unilateral total mastectomy for breast malignancy followed by immediate breast reconstruction with a silicone implant, either in the subpectoral or prepectoral plane, at Yeungnam University Hospital between October 2012 and December 2022. Only patients who underwent VECTRA XT 3D scans more than one year after surgery were included. Patients who simultaneously underwent other cosmetic breast surgeries, such as augmentation, or those with grade 3 ptosis (according to Regnault), as well as patients who had minimal invasive and robotic mastectomy, were excluded.

In a multidisciplinary meeting, a comprehensive discussion was conducted regarding most of the patients, with a focus on the resections for breast cancer performed at the Breast and Endocrine Surgery Department of Yeungnam University Hospital. Most patients underwent nipple-sparing mastectomy using the horizontal radial incision technique, with only one patient receiving a periareolar incision with lateral extension. No cases involved the inframammary fold incision. The choice of incision types was meticulously determined according to the specific location and extent of the cancer to optimize surgical outcomes, with all reconstructions carried out by two plastic surgeons, J.H.L. and I.K.K.

In this retrospective study, 63 patients participated, with 29 in the subpectoral group and 38 in the prepectoral group. The patient characteristics documented from medical records included age, body mass index (BMI; kg/m^2^), follow-up duration, tumor laterality, cancer stage, and adjuvant chemotherapy or radiotherapy history. The operation-related characteristics included mastectomy weight and volume, breast implant volume, and the surface area of ADM used.

**Figure 1:**
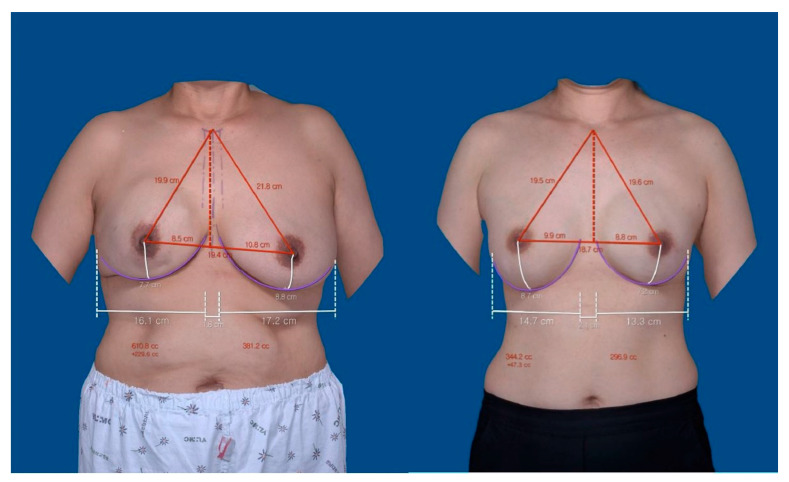
One-year postoperative VECTRA XT 3D scanning images. (Left) Right breast cancer patient who underwent subpectoral direct-to-implant (DTI). (Right) Left breast cancer patient who underwent prepectoral DTI. (**Left**) Subpectoral DTI; (**Right**) Prepectoral DTI. The sternal notch (SN) and both nipples (N) form a triangle (red line) along with the midline (ML, red dotted line). The inframammary fold (IMF, purple curved line), medial mammary fold to lateral mammary fold (MMF to LMF; breast width, white horizontal line), and breast volume are also shown.

Silicone implants from various manufacturers, including BIOCELL, Allergan, Bellagel, EUROSILICONE, Sebbin, and Mentor, were used. ADM products such as CGCryoDerm (CGBio, Seoul, Republic of Korea), Megaderm (L&C Bio, Seoul, Republic of Korea), and MyDerm (MSBIO, Seongnam-si, Republic of Korea) were used in all cases. For subpectoral implants, ADM was used to cover the lower pole of the implant, while for prepectoral implants, ADM was applied using a total wrapping method, providing both anterior and posterior coverage [32]. All patients visited the outpatient clinic one year after surgery, where they underwent VECTRA XT 3D scanning with arms placed at their sides. The scans were analyzed using software to establish landmarks including the nipple (N), sternal notch (SN), midclavicular line (MC), midline (ML), inframammary fold (IMF), and medial/lateral mammary folds (MMFs/LMFs). These landmarks were automatically marked as points upon scanning by a single plastic surgeon, and if there is a significant error in the location, each landmark can be manually adjusted. Afterward, by pressing the print button in the software, various measurements, including the distance between landmarks, can be viewed. Measurements were taken for the SN–N distance (SN-N), ML–N distance (ML-N), N–IMF distance (N-IMF), breast width (MMF–LMF distance), and breast volume of each breast. Differences between the reconstructed and contralateral breasts were assessed, and these values’ ratios were calculated. Additionally, breast projection differences between both breasts were measured (Figure 1). We also examined the impacts of BMI and age on the aforementioned measurements. Based on the World Health Organization (WHO) standards, a BMI of 25 or greater is classified as pre-obese, so we used a BMI of 25 as the threshold value. Furthermore, considering that the average menopausal age in the Republic of Korea is around 50 years [33], we used 50 years as the threshold for age group classification.

### 2.2. Statistical Analysis

Data analyses were conducted utilizing SPSS software (version 27; IBM, Armonk, NY, USA). Categorical variables are expressed as counts and percentages (%), while continuous variables are reported as the mean ± standard deviation. The chi-square test was used to assess differences in proportions between the groups. Furthermore, the mean differences between groups were evaluated through a two-sample *t*-test, which was applied based on the normality of the data distribution. A *p*-value of less than 0.05 was considered statistically significant.

## 3. Results

### 3.1. Patient Demographics

A comparison between the subpectoral and prepectoral groups revealed that, except for the follow-up period and the surface area of the inserted ADM, no other variables showed statistically significant differences between the two groups. Specifically, the follow-up periods were 55.0 ± 25.3 months for the subpectoral group and 30.9 ± 15.3 months for the prepectoral group, respectively (*p* < 0.001). Additionally, the surface areas of the inserted ADM were 117.4 ± 45.1 cm^2^ for the subpectoral group and 264.2 ± 99.0 cm^2^ for the prepectoral group, respectively (*p* < 0.001) (Table 1). Neither group included participants who underwent neoadjuvant chemotherapy.

### 3.2. Morphological Breast Shape Measurements Between Subpectoral and Prepectoral Groups

The results of the breast shape ratio, calculated using the measured SN-N, ML-N, N-IMF, breast width, breast volume, and differences in breast projection between the reconstructed and contralateral breasts, are presented in Table 2. The SN-N ratio was 0.91 ± 0.06 for the subpectoral group and 0.95 ± 0.06 for the prepectoral group, with the prepectoral group having a value closer to 1, which was statistically significant (*p* = 0.014). Other measurements showed no statistically significant differences between these groups.

### 3.3. Evaluation of Breast Shape Ratio Based on BMI and Age Between Subpectoral and Prepectoral Groups

Based on BMI and age, differences in measurements between the subpectoral and prepectoral groups are presented in Table 3 and Table 4. When categorizing patients by a BMI threshold of 25, the SN-N ratio was 0.89 ± 0.06 in the subpectoral group and 0.97 ± 0.07 in the prepectoral group for those with a BMI of 25 or higher, with the prepectoral group being closer to 1 and showing a statistically significant difference (*p* = 0.027). In contrast, for patients with a BMI below 25, no statistically significant difference was observed between the two groups. Other measurements did not show statistically significant differences between the groups according to BMI. Regarding age, when categorized at 50 years, no measurements showed statistically significant differences between the two groups based on the surgical method.

## 4. Discussion

In IBBR, the prepectoral approach confers several benefits, including reduced distortion of breast projection, prevention of animation deformity caused by pectoralis major muscle contraction, and the attainment of a more natural, ptotic contour lower on the chest wall compared to subpectoral placement. These factors contribute to potentially better aesthetic outcomes by reducing device malposition caused by muscle foreshortening, as evidenced by studies indicating improved breast projection, a reduced incidence of muscle animation, and restoration of a more natural breast shape [11,31,32]. However, this approach has its drawbacks, including the higher cost of ADM and potential issues such as superior pole implant visibility, palpability, and rippling [34]. Both techniques have respective advantages and disadvantages, and numerous studies have compared their aesthetic and surgical outcomes. For instance, a study that compared 112 cases of prepectoral DTI breast reconstruction with a historical subpectoral cohort found that the prepectoral approach had a lower incidence of partial thickness necrosis and fewer aesthetic revisions [28]. Another study evaluating aesthetic outcomes utilizing the BREAST-Q for subjective satisfaction, along with a Likert scale for assessing rippling, implant visibility, and palpability, concluded that the prepectoral group generally demonstrated superior results [35]. Although the occurrence of rippling deformities was more prevalent in the prepectoral group, this approach was linked to reduced animation deformities and a lower rate of Baker grade III capsular contracture, suggesting a potentially more favorable overall aesthetic outcome [14,36].

Despite these findings, studies specifically evaluating asymmetry after IBBR are limited [37,38], and none have utilized VECTRA XT 3D for comparing the subpectoral and prepectoral planes of IBBR. Previous studies have used various methods to assess asymmetry. One study compared the asymmetry between subpectoral and prepectoral groups using BCCT.core software version 3.0 to measure SN-N discrepancy and breast retraction assessment (BRA) both preoperatively and postoperatively. The prepectoral group demonstrated a more pronounced increase in asymmetry; however, no statistically significant difference in postoperative asymmetry was identified between the two techniques [39]. In a study of 26 patients who underwent DTI breast reconstruction, postoperative outcomes such as symmetry were assessed one year after surgery. Clinical photographs were evaluated from multiple frontal, lateral, and oblique views by three plastic surgeons and three general practitioners. The results indicated no significant differences in postoperative outcomes between the two surgical techniques [40].

Various landmarks and measurements have been used to evaluate breast asymmetry. In one study, a 3D Vectra Camera (Canfield Scientific Inc., Parsippany, NJ, USA) was used to measure SN-N, N-IMF, ML-N, and breast width in 100 healthy women, emphasizing that SN-N and ML-N measurements form the aesthetic triangle of the breast, highlighting their significance [22]. Another study used 3D image scanning to evaluate breast asymmetry in breast cancer patients by comparing preoperative SN-N and volume ratios, considering absolute differences greater than 5 mm in SN-N and 50 mL in volume as indicative of asymmetry [41]. Moreover, when comparing breast shape changes one-year postoperatively based on the ADM placement method in DTI breast reconstruction, the ratio [(follow-up distance − initial distance)/initial distance] was used for SN-N and MC-N measurements [42].

Breast asymmetry can be influenced by various factors. A study involving 42 patients who underwent augmentation mammaplasty with implants found that implant rotation and mobility led to ptosis and changes in the level of the inframammary fold, contributing to asymmetry [43]. Intrinsic factors, such as genetic predisposition, tissue atrophy, weight changes related to sagging, and hormonal changes due to pregnancy and lactation after surgery, also play significant roles [44,45]. A study using multiple linear regression analysis and logistic regression models on patients with breast cancer found that BMI, age, and cancer status did not significantly impact the SN-N ratio and volume ratio. However, a higher BMI was associated with an increased likelihood of ptosis disagreement, and being of Caucasian ethnicity contributed to a better breast volume ratio [41].

Tools for evaluating breast asymmetry can be broadly categorized as subjective (perception) and objective (measurement). Many studies have utilized the BREAST-Q™ questionnaire during the postoperative period as a patient-reported outcome measurement (PROM) to assess not only the quality of life but also satisfaction with aesthetic results [46,47,48]. However, its application is limited in evaluating specific patient situations and needs [49]. Therefore, more objective assessment tools have been developed, including BCCT.core3^®^, BAT^®^, OBCS^®^, and GBAI^®^. These tools calculate asymmetry scales based on landmarks, such as the sternal notch and nipple position, utilizing measurements of distances, areas, and skin color [50,51,52,53]. However, these methods are limited by their reliance on frontal images for calculation, which may affect the accuracy of measurements. Therefore, the VECTRA XT 3D, a 3D imaging system, has recently been introduced for measuring asymmetry. One study validated its accuracy for assessing breast volume and shape symmetry after reconstruction using a breast phantom model. The study noted its potential for evaluating aesthetic outcomes due to minimal observer variation and high reproducibility [25].

This study used VECTRA XT 3D scanning to compare the asymmetry of subpectoral and prepectoral DTI breast reconstructions based on various measurements. Among the multiple landmarks evaluated, significant differences were found in the SN-N ratio, which is crucial for determining the nipple position and contributing to breast symmetry. The SN-N ratio in the prepectoral group was closer to 1, indicating better symmetry between both breasts. Additionally, when comparing measurements between the subpectoral and prepectoral groups based on BMI and age at the time of surgery, significant differences were found only in the SN-N ratio. For patients with a BMI of 25 or higher, the SN-N ratio was closer to 1 in the prepectoral group; for patients with a BMI below 25, the SN-N ratio was 0.92 ± 0.06 for the subpectoral group and 0.94 ± 0.05 for the prepectoral group (*p* = 0.193). These findings showed differences in nipple positioning between the subpectoral and prepectoral groups. Given that there were no statistically significant differences in breast width or volume between the two groups, it can be concluded that the variations in asymmetry stem from differences in nipple positioning related to the use of either the subpectoral or prepectoral technique in DTI breast reconstruction. The superior symmetry observed with the prepectoral approach can be attributed to the preservation of natural breast tissue and its inherent structural support, which is often compromised to a greater extent in the subpectoral technique. This preservation not only facilitates a more aesthetically pleasing outcome but also maintains the integrity of the breast contour, ultimately enhancing overall symmetry in breast reconstruction procedures. Therefore, the advantages of employing the prepectoral technique are underscored by its capacity to retain essential anatomical features vital for achieving optimal aesthetic results. Notably, the increased symmetry observed in patients with a higher BMI when utilizing the prepectoral technique can be attributed to the greater volume of subcutaneous tissue in these individuals, which enhances the likelihood of a naturally ptotic breast shape preoperatively. This natural ptotic contour likely contributes to the improved results achieved with the prepectoral technique, effectively enhancing this shape.

Aesthetic results after breast reconstruction are crucial for patient satisfaction. While such outcomes can be assessed through surveys, numerical measurements can provide objectivity. This study used the VECTRA XT 3D imaging system to obtain various measurements related to aesthetic outcomes with high accuracy, thereby enhancing objectivity. The 3D imaging technology also compensated for the inaccuracies inherent in traditional 2D images and clinical measurement, allowing for a reliable comparison between subpectoral and prepectoral DTI breast reconstruction. Given that breast symmetry can change over time following IBBR due to factors such as breast sagging and changes in elasticity, and noting that photographic analysis of prepectoral IBBR patients showed a decrease in breast symmetry from 84.4% preoperatively to 56.7% at 2–5 years postoperatively [54], our study is also significant for the long-term observation of its postoperative asymmetry.

Based on the findings of this study, VECTRA XT 3D allows for highly accurate measurements of breast shape symmetry after IBBR and can identify factors influencing symmetry for individual patients by using various landmarks and indicators within the software. Additionally, as the number of patient cases increases, predictive models for changes in symmetry indicators could be developed through statistical analysis. These would help in establishing patient-specific surgical approaches [55]. While additional research involving patients with varied conditions is necessary, the findings of this study suggest that, particularly in individuals with a high BMI, prepectoral DTI provides superior symmetry compared to subpectoral DTI. Therefore, prepectoral DTI can be recommended when possible. In other patients, if potential complications are well understood, there is no significant difference in symmetry between subpectoral and prepectoral DTI. Thus, this information can help guide surgical decisions based on the patient’s individual needs and preferences and will be useful for surgeons in their planning.

This study has several limitations. According to one study, when measuring breast asymmetry, the nipple position and its difference are crucial, with MC-N and SN-N being important landmarks [56]. MC-N has been suggested as a superior landmark for assessing correct nipple positioning compared to SN-N. However, this study did not measure MC-N. Furthermore, the limited sample size inherently constrains the generalizability of the findings, possibly restricting their applicability to a broader population. Compounding these issues is the monocentric nature of the case series, which introduces an additional layer of limitation. Furthermore, since direct-to-implant (DTI) breast reconstructions were conducted by two different plastic surgeons, variations in surgical techniques and approaches, such as differences in the surgical technique of wrapping the ADM during prepectoral breast reconstruction, could contribute to discrepancies in outcomes, affecting the consistency and reliability of the results.

## 5. Conclusions

Postoperative asymmetry is common in IBBR. Although both subpectoral and prepectoral techniques are used, their long-term aesthetic outcomes have not been objectively compared. This study is the first to use VECTRA XT 3D scanning to quantitatively measure and assess breast asymmetry between the subpectoral and prepectoral techniques, revealing that the prepectoral technique results in better nipple positioning and overall symmetry compared to the subpectoral technique. VECTRA XT 3D, with its higher accuracy compared to other methods, can be a useful tool for evaluating aesthetic outcomes and monitoring postoperative progress. To further understand the factors and indicators influencing breast symmetry after IBBR based on individual patient characteristics, multicenter studies with larger patient populations are needed. Additionally, the development of predictive models using statistical analysis could be beneficial. These advancements could help guide patients in their choice of surgical method and assist surgeons in their surgical planning.

## Figures and Tables

**Table 1:** Patient demographics: subpectoral vs. prepectoral.

Variable	Subpectoral	Prepectoral	*p*-Value
No. of patients (%)	29 (43.3)	38 (56.7)	
Age, mean ± SD (years)	51.5 ± 6.9	48.1 ± 7.2	0.056
BMI, mean ± SD (kg/m^2^)	23.8 ± 3.8	22.8 ± 3.0	0.261
Adjuvant chemotherapy (%)	13 (43.3)	17 (56.7)	0.250
Adjuvant radiotherapy (%)	2 (100)	0	0.100
Follow-up duration, mean ± SD (months)	55.0 ± 25.3	30.9 ± 15.3	<0.001 ^a^
Laterality of cancer			0.180
Right side: No. (%)	12 (35.3)	22 (64.7)	
Left side: No (%)	17 (51.5)	16 (48.5)	
Cancer stage			0.623
No. of stage 0 (%)	6 (40)	9 (60)	
No. of stage I (%)	13 (39.4)	20 (60.6)	
No. of stage II (%)	10 (52.6)	9 (47.4)	
Mastectomy specimen weight, mean ± SD (g)	277.6 ± 118.8	258.6 ± 104.4	0.489
Mastectomy specimen volume, mean ± SD (cc)	251.4 ± 115.7	239.47 ± 111.7	0.671
Inserted implant volume, mean ± SD (cc)	238.0 ± 87.9	228.6 ± 75.9	0.640
Surface area of inserted ADM, mean ± SD (cm^2^)	117.4 ± 45.1	264.2 ± 99.0	<0.001 ^a^

^a^ *p* < 0.05. BMI, body mass index; ADM, acellular dermal matrix.

**Table 2:** Morphological breast shape measurement between subpectoral and prepectoral breast reconstruction group.

	Subpectoral			Prepectoral			
Variable	Reconstructed Breast (a)	Contralateral Breast (b)	Ratio (a/b)	Reconstructed Breast (a)	Contralateral Breast (b)	Ratio (a/b)	*p*-Value
SN-N, cm	19.3 ± 1.6	21.3 ± 2.1	0.91 ± 0.06	20.1 ± 1.8	21.3 ± 2.3	0.95 ± 0.06	0.014 ^a^
ML-N, cm	8.8 ± 1.0	9.4 ± 1.3	0.95 ± 0.18	9.0 ± 1.5	9.8 ± 1.3	0.95 ± 0.20	0.707
N-IMF, cm	6.9 ± 1.4	6.5 ± 1.3	1.07 ± 0.21	6.7 ± 1.1	6.6 ± 1.1	1.02 ± 0.16	0.255
Breast width, cm	14.3 ± 1.7	14.9 ± 1.7	0.96 ± 0.09	14.1 ± 1.9	14.8 ± 1.9	0.96 ± 0.11	0.973
Breast volume, cc	253.3 ± 113.3	277.6 ± 152.3	0.99 ± 0.31	263.8 ± 136.1	263.9 ± 147.7	1.12 ± 0.59	0.262
Breast projection difference, cm	0.54 ± 0.45			0.65 ± 0.48			0.355

All values are presented as the mean ± standard deviation (SD). ^a^ *p* < 0.05. SN-N, sternal-notch-to-nipple distance; ML-N, midline-to-nipple distance; N-IMF, nipple-to-inframammary fold distance.

**Table 3:** Comparison of breast shape average ratio based on BMI: subpectoral vs. prepectoral.

	BMI < 25 (*n* = 48)			BMI ≥ 25 (*n* = 19)		
Variable	Subpectoral (*n* = 17)	Prepectoral (*n* = 31)	*p*-Value	Subpectoral (*n* = 12)	Prepectoral (*n* = 7)	*p*-Value
SN-N ratio	0.92 ± 0.06	0.94 ± 0.06	0.193	0.89 ± 0.06	0.97 ± 0.07	0.027 ^a^
N-IMF ratio	1.11 ± 0.19	1.00 ± 0.16	0.063	1.03 ± 0.23	1.09 ± 0.13	0.509
ML-N ratio	1.01 ± 0.20	0.92 ± 0.18	0.141	0.88 ± 0.13	0.99 ± 0.26	0.194
Breast width ratio	0.94 ± 0.08	0.94 ± 0.10	0.980	0.98 ± 0.09	1.03 ± 0.09	0.263
Breast volume ratio	1.04 ± 0.29	1.09 ± 0.59	0.742	0.92 ± 0.32	1.28 ± 0.57	0.092
Breast projection difference, cm	0.58 ± 0.44	0.64 ± 0.44	0.674	0.48 ± 0.47	0.68 ± 0.66	0.449

All values are presented as the mean ± standard deviation (SD). ^a^ *p* < 0.05. BMI, body mass index; SN-N, sternal-notch-to-nipple distance; ML-N, midline-to-nipple distance; N-IMF, nipple-to-inframammary fold distance.

**Table 4:** Comparison of breast shape average ratio based on age: subpectoral vs. prepectoral.

	Age < 50 (*n* = 34)			Age ≥ 50 (*n* = 33)		
Variable	Subpectoral (*n* = 11)	Prepectoral (*n* = 23)	*p*-Value	Subpectoral (*n* = 18)	Prepectoral (*n* = 15)	*p*-Value
SN-N ratio	0.94 ± 0.31	0.96 ± 0.06	0.336	0.89 ± 0.06	0.93 ± 0.07	0.09
N-IMF ratio	1.15 ± 0.18	1.05 ± 0.17	0.106	1.02 ± 0.21	0.98 ± 0.14	0.502
ML-N ratio	0.93 ± 0.13	0.96 ± 0.17	0.594	0.96 ± 0.21	0.88 ± 0.23	0.325
Breast width ratio	0.96 ± 0.09	0.95 ± 0.12	0.870	0.96 ± 0.09	0.98 ± 0.14	0.730
Breast volume ratio	1.03 ± 0.32	1.20 ± 0.73	0.475	0.96 ± 0.31	1.01 ± 0.23	0.611
Breast projection difference, cm	0.48 ± 0.47	0.68 ± 0.43	0.227	0.58 ± 0.44	0.60 ± 0.56	0.896

All values are presented as the mean ± standard deviation (SD). BMI, body mass index; SN-N, sternal-notch-to-nipple distance; ML-N, midline-to-nipple distance; N-IMF, nipple-to-inframammary fold distance.

## Data Availability

The data presented in this study are available on request from the corresponding author. The data are not publicly available due to consideration for the patients’ privacy.

## References

[1] Nahabedian M.Y. (2016). Implant-based breast reconstruction: Strategies to achieve optimal outcomes and minimize complications. J. Surg. Oncol..

[2] Lohmander F., Lagergren J., Johansson H., Roy P.G., Frisell J., Brandberg Y. (2020). Quality of life and patient satisfaction after implant-based breast reconstruction with or without acellular dermal matrix: Randomized clinical trial. BJS Open.

[3] Colwell A.S., Taylor E.M. (2020). Recent Advances in Implant-Based Breast Reconstruction. Plast. Reconstr. Surg..

[4] Cordeiro P.G., McCarthy C.M. (2006). A single surgeon’s 12-year experience with tissue expander/implant breast reconstruction: Part I. A prospective analysis of early complications. Plast. Reconstr. Surg..

[5] Hammond D.C., Schmitt W.P., O’Connor E.A. (2015). Treatment of breast animation deformity in implant-based reconstruction with pocket change to the subcutaneous position. Plast. Reconstr. Surg..

[6] Graziano F.D., Lu J., Sbitany H. (2023). Prepectoral Breast Reconstruction. Clin. Plast. Surg..

[7] Bekisz J.M., Salibian A.A., Frey J.D., Choi M., Karp N.S. (2022). Picking the Right Plane: A Comparison of Total Submuscular, Dual-Plane, and Prepectoral Implant-Based Breast Reconstruction. Plast. Reconstr. Surg..

[8] Ozgur I., Kurul S., Bademler S., Karanlik H. (2021). Comparison of subpectoral versus dual-plane implant based immediate breast reconstruction after nipple-areola sparing mastectomy. Ann. Chir. Plast. Esthet..

[9] Vardanian A.J., Clayton J.L., Roostaeian J., Shirvanian V., Da Lio A., Lipa J.E., Crisera C., Festekjian J.H. (2011). Comparison of implant-based immediate breast reconstruction with and without acellular dermal matrix. Plast. Reconstr. Surg..

[10] Salibian A.A., Karp N.S. (2023). Modern Approaches to Implant-Based Breast Reconstruction. Clin. Plast. Surg..

[11] Sigalove S., Maxwell G.P., Sigalove N.M., Storm-Dickerson T.L., Pope N., Rice J., Gabriel A. (2017). Prepectoral Implant-Based Breast Reconstruction: Rationale, Indications, and Preliminary Results. Plast. Reconstr. Surg..

[12] Wazir U., Mokbel K. (2018). The evolving role of pre-pectoral ADM-assisted implant-based immediate breast reconstruction following skin-sparing mastectomy. Am. J. Surg..

[13] Lee J.H., Choi B.G., Lee W.S., Seo M.G., Park B.Y., Kim Y.S., Park D.Y., Kim I.K. (2023). Long-Term Ultrasonographic and Histologic Changes in Acellular Dermal Matrix in Implant-Based Breast Reconstructions. Plast. Reconstr. Surg..

[14] Franceschini G., Scardina L., Di Leone A., Terribile D.A., Sanchez A.M., Magno S., D’Archi S., Franco A., Mason E.J., Carnassale B. (2021). Immediate Prosthetic Breast Reconstruction after Nipple-Sparing Mastectomy: Traditional Subpectoral Technique versus Direct-to-Implant Prepectoral Reconstruction without Acellular Dermal Matrix. J. Pers. Med..

[15] Salgarello M., Fabbri M., Visconti G., Barone Adesi L. (2024). Implant-Based Breast Reconstruction After Nipple-Sparing and Skin-Sparing Mastectomy in Breast-Augmented Patients: Prepectoral or Submuscular Direct-to-Implant Reconstruction?. Aesthet. Surg. J..

[16] Belmonte B.M., Campbell C.A. (2021). Safety Profile and Predictors of Aesthetic Outcomes After Prepectoral Breast Reconstruction with Meshed Acellular Dermal Matrix. Ann. Plast. Surg..

[17] Guyomard V., Leinster S., Wilkinson M. (2007). Systematic review of studies of patients’ satisfaction with breast reconstruction after mastectomy. Breast.

[18] Fang S.Y., Shu B.C., Chang Y.J. (2013). The effect of breast reconstruction surgery on body image among women after mastectomy: A meta-analysis. Breast Cancer Res. Treat..

[19] Qureshi A.A., Odom E.B., Parikh R.P., Myckatyn T.M., Tenenbaum M.M. (2017). Patient-Reported Outcomes of Aesthetics and Satisfaction in Immediate Breast Reconstruction After Nipple-Sparing Mastectomy With Implants and Fat Grafting. Aesthet. Surg. J..

[20] Sampathkumar U., Bui T., Liu J., Nowroolizarki Z., Bordes M.C., Hanson S.E., Reece G.P., Markey M.K., Merchant F.A. (2023). Objective Analysis of Breast Symmetry in Female Patients Undergoing Breast Reconstruction After Total Mastectomy. Aesthet. Surg. J. Open Forum.

[21] Tebbetts J.B. (2011). Correction of breast asymmetry does not exist, and the role of three-dimensional imaging remains a question. Plast. Reconstr. Surg..

[22] Henseler H. (2023). Exploring natural breast symmetry in the female plastic surgical patient population. GMS Interdiscip. Plast. Reconstr. Surg. DGPW.

[23] Godden A.R., O’Connell R.L., Barry P.A., Krupa K.C.D., Wolf L.M., Mohammed K., Kirby A.M., Rusby J.E. (2020). 3-Dimensional objective aesthetic evaluation to replace panel assessment after breast-conserving treatment. Breast Cancer.

[24] Pham M., Alzul R., Elder E., French J., Cardoso J., Kaviani A., Meybodi F. (2023). Evaluation of Vectra^®^ XT 3D Surface Imaging Technology in Measuring Breast Symmetry and Breast Volume. Aesthetic Plast. Surg..

[25] O’Connell R.L., Khabra K., Bamber J.C., deSouza N., Meybodi F., Barry P.A., Rusby J.E. (2018). Validation of the Vectra XT three-dimensional imaging system for measuring breast volume and symmetry following oncological reconstruction. Breast Cancer Res. Treat..

[26] Li L., Su Y., Xiu B., Huang X., Chi W., Hou J., Zhang Y., Tian J., Wang J., Wu J. (2019). Comparison of prepectoral and subpectoral breast reconstruction after mastectomies: A systematic review and meta analysis. Eur. J. Surg. Oncol..

[27] Kim J.H., Hong S.E. (2020). A Comparative Analysis between Subpectoral versus Prepectoral Single Stage Direct-to-Implant Breast Reconstruction. Medicina.

[28] Mirhaidari S.J., Azouz V., Wagner D.S. (2020). Prepectoral Versus Subpectoral Direct to Implant Immediate Breast Reconstruction. Ann. Plast. Surg..

[29] Cogliandro A., Salzillo R., De Bernardis R., Loria F.S., Petrucci V., Barone M., Tenna S., Cagli B., Persichetti P. (2023). Prepectoral Versus Subpectoral Direct-to-Implant Breast Reconstruction: Evaluation of Patient’s Quality of Life and Satisfaction with BREAST-Q. Aesthetic Plast. Surg..

[30] Ostapenko E., Nixdorf L., Devyatko Y., Exner R., Wimmer K., Fitzal F. (2023). Prepectoral Versus Subpectoral Implant-Based Breast Reconstruction: A Systemic Review and Meta-analysis. Ann. Surg. Oncol..

[31] Piccolo P.P., Venturi M., Mesbahi A.N., Nahabedian M.Y. (2023). Current status prepectoral and subpectoral breast reconstruction in the USA. Gland. Surg..

[32] Sohn S.M., Lee H.C., Park S.H., Yoon E.S. (2023). Difference in the outcomes of anterior tenting and wrapping techniques for acellular dermal matrix coverage in prepectoral breast reconstruction. J. Plast. Reconstr. Aesthet. Surg..

[33] Shin Y.J., Song J.Y., Kim M.J., Choi J.I., Han K.D., Lee H.N. (2017). Relationship between age at last delivery and age at menopause: The Korea National Health and Nutrition Examination Survey. Obstet. Gynecol. Sci..

[34] Highton L., Johnson R., Kirwan C., Murphy J. (2017). Prepectoral Implant-Based Breast Reconstruction. Plast. Reconstr. Surg. Glob. Open.

[35] Bernini M., Calabrese C., Cecconi L., Santi C., Gjondedaj U., Roselli J., Nori J., Fausto A., Orzalesi L., Casella D. (2015). Subcutaneous Direct-to-Implant Breast Reconstruction: Surgical, Functional, and Aesthetic Results after Long-Term Follow-Up. Plast. Reconstr. Surg. Glob. Open.

[36] Yang J.Y., Kim C.W., Lee J.W., Kim S.K., Lee S.A., Hwang E. (2019). Considerations for patient selection: Prepectoral versus subpectoral implant-based breast reconstruction. Arch. Plast. Surg..

[37] Cohen O., Small K., Lee C., Petruolo O., Karp N., Choi M. (2016). Is Unilateral Implant or Autologous Breast Reconstruction Better in Obtaining Breast Symmetry?. Breast J..

[38] Huang D.W., Chou Y.Y., Liu H.H., Dai N.T., Tzeng Y.S., Chen S.G. (2022). Is 3-Dimensional Scanning Really Helpful in Implant-Based Breast Reconstruction? A Prospective Study. Ann. Plast. Surg..

[39] Mercury O., Nores G.G., Carlson G.W. (2022). Symmetry of Nipple Position After Bilateral Nipple-Sparing Mastectomy and Implant-Based Reconstruction: The Impact of Reconstructive Method. Ann. Plast. Surg..

[40] Grippaudo F.R., Ribuffo D. (2023). Prepectoral Versus Subpectoral Implant-based Breast Reconstruction: Evaluation of the Aesthetic Outcomes by Plastic Surgeons and General Practitioners. Aesthetic Med..

[41] Cheong A.L., Liu J., Reece G.P., Nicklaus K.M., Catherine Bordes M., Hanson S.E., Markey M.K., Merchant F.A. (2019). Natural Breast Symmetry in Preoperative Breast Cancer Patients. Plast. Reconstr. Surg. Glob. Open.

[42] Han W.Y., Han S.J., Eom J.S., Kim E.K., Han H.H. (2023). A Comparative Study of Wraparound versus Anterior Coverage Placement of Acellular Dermal Matrix in Prepectoral Breast Reconstruction. Plast. Reconstr. Surg..

[43] Patlazhan G., Shkolnaya O., Torubarov I., Gomes M. (2020). Our 10 Years’ Experience in Breast Asymmetry Correction. Aesthetic Plast. Surg..

[44] Reilley A.F. (2006). Breast asymmetry: Classification and management. Aesthet. Surg. J..

[45] Stahl S., Dannehl D., Daigeler A., Jorge C., Brendlin A., Hagen F., Santos Stahl A., Feng Y.S., Nikolaou K., Estler A. (2023). Definitions of Abnormal Breast Size and Asymmetry: A Cohort Study of 400 Women. Aesthetic Plast. Surg..

[46] Arora N., Patel R., Sohi G., Merchant S., Martou G. (2023). A Scoping Review of the Application of BREAST-Q in Surgical Research. JPRAS Open.

[47] Wampler A.T., Powelson I.A., Homa K., Freed G.L. (2021). BREAST-Q Outcomes before and after Bilateral Reduction Mammaplasty. Plast. Reconstr. Surg..

[48] Seth I., Seth N., Bulloch G., Rozen W.M., Hunter-Smith D.J. (2021). Systematic Review of Breast-Q: A Tool to Evaluate Post-Mastectomy Breast Reconstruction. Breast Cancer Targets Ther..

[49] Liu L.Q., Branford O.A., Mehigan S. (2018). BREAST-Q Measurement of the Patient Perspective in Oncoplastic Breast Surgery: A Systematic Review. Plast. Reconstr. Surg. Glob. Open.

[50] Monton J., Torres A., Gijon M., Chang-Azancot L., Kenig N., Trandafir P.C., Jordan J., Insausti R. (2020). Use of Symmetry Assessment Methods in the Context of Breast Surgery. Aesthetic Plast. Surg..

[51] Yu T., Eom K.Y., Jang N.Y., Kim K.S., Koo T.R., Kwon J., Kim B.H., Kang E., Kim S.W., Kim J.S. (2016). Objective Measurement of Cosmetic Outcomes of Breast Conserving Therapy Using BCCT.core. Cancer Res. Treat..

[52] Krois W., Romar A.K., Wild T., Dubsky P., Exner R., Panhofer P., Jakesz R., Gnant M., Fitzal F. (2017). Objective breast symmetry analysis with the breast analyzing tool (BAT): Improved tool for clinical trials. Breast Cancer Res. Treat..

[53] Soror T., Lancellotta V., Kovács G., Lanzotti V., Tagliaferri L., Casà C., Aristei C., Barberini F., Mahmoud M., Badakhshi H. (2020). kOBCS(©): A novel software calculator program of the Objective Breast Cosmesis Scale (OBCS). Breast Cancer.

[54] Nahabedian M.Y. (2020). What Are the Long-Term Aesthetic Issues in Prepectoral Breast Reconstruction?. Aesthet. Surg. J..

[55] Lee S.O., Lee J.H. (2024). Prediction model for intraoperative implant volume using the 3D surface imaging system (VECTRA XT 3D) in direct-to-implant breast reconstructions. Gland. Surg..

[56] Kim J.Y.S., Qiu C.S., Chiu W.K., Feld L.N., Mioton L.M., Kearney A., Fracol M. (2019). A Quantitative Analysis of Animation Deformity in Prosthetic Breast Reconstruction. Plast. Reconstr. Surg..

